# The Therapeutic Effect of Traditional Chinese Medicine on Inflammatory Diseases Caused by Virus, Especially on Those Caused by COVID-19

**DOI:** 10.3389/fphar.2021.650425

**Published:** 2021-05-26

**Authors:** Peng Li, Shuang Hu, Cheng Qian, Yan Yao, Liang-yun Li, Jun-fa Yang, Li Yang, Chen-chen Yang, Hong Zhou, Shu-xian Wang, Ying Hu, Xing-yu Zhu, Jing Zhou, Lin-xin Pan, Chuan-pu Shen, Huan Zhou

**Affiliations:** ^1^ The First Affiliated Hospital of Medical University of Anhui, Hefei, China; ^2^ School of Pharmacy, Anhui Key Laboratory of Bioactivity of Natural Products, Anhui Medical University, Hefei, China; ^3^ Institute for Liver Diseases of Anhui Medical University, Anhui Medical University, Hefei, China; ^4^ Center for Scientific Research, Anhui Medical University, Hefei, China; ^5^ National Drug Clinical Trial Institution, The First Affiliated Hospital of Bengbu Medical College, Hefei, China; ^6^ School of Life Sciences, Anhui Medical University, Hefei, China

**Keywords:** COVID-19, TCM, inflammatory reaction, TCM formulation, therapeutic targets

## Abstract

Inflammasomes are large multimolecular complexes best recognized because of their ability to control activation of caspase-1, which in turn regulates the maturation of interleukin-18 (IL-18) and interleukin-1 β (IL-1β). IL-1β was originally identified as a pro-inflammatory cytokine, capable of inducing local and systemic inflammation as well as a fever response reaction in response to infection or injury. Excessive production of IL-1β is related to inflammatory and autoimmune diseases. Both coronavirus disease 2019 (COVID-19) and severe acute respiratory syndrome (SARS) are characterized by excessive inflammatory response. For SARS, there is no correlation between viral load and worsening symptoms. However, there is no specific medicine which is available to treat the disease. As an important part of medical practice, TCM showed an obvious therapeutic effect in SARS-CoV-infected patients. In this article, we summarize the current applications of TCM in the treatment of COVID-19 patients. Herein, we also offer an insight into the underlying mechanisms of the therapeutic effects of TCM, as well as introduce new naturally occurring compounds with anti-coronavirus activity, in order to provide a new and potential drug development strategy for the treatment of COVID-19.

## Introduction

**FIGURE 1:**
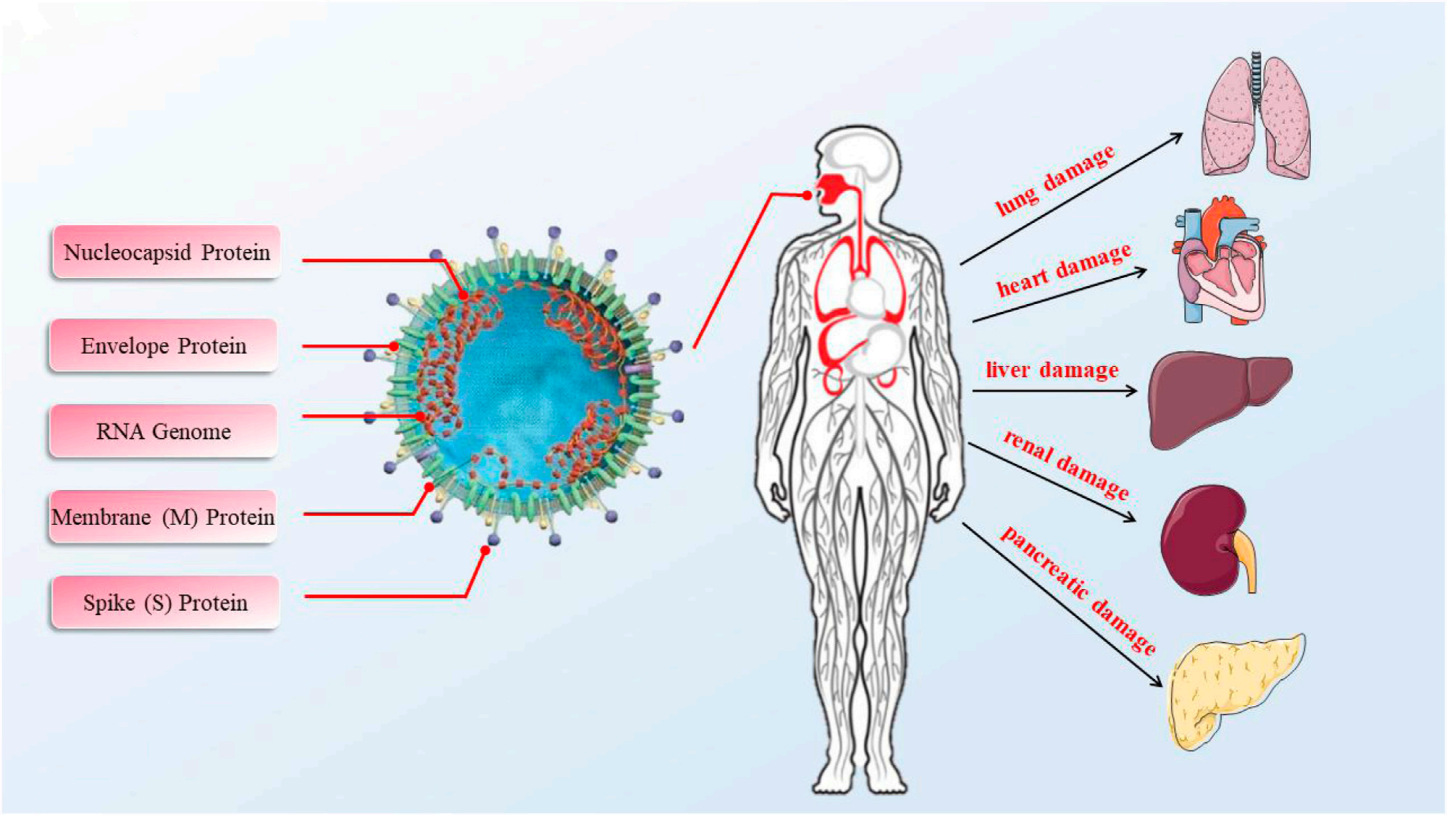
The virus structure of SARS-CoV-2 and its organ damage. It was found that in the 52 COVID-19 patients, the incidence of heart injury was 33% (abnormal LDH or creatine kinase), liver injury was 29% (any abnormality in AST, ALT, GGT, or ALP), pancreatic injury was 17%, renal injury was 8% (abnormal creatinine), and diarrhea was 2%.

Viruses cause a wide range of human diseases, ranging from acute self-healing conditions to acute fatal diseases. Effects that appear long after the primary infection may also increase the incidence of chronic diseases or lead to the development of cancer [25366544. Coronaviruses belong to the subfamily Orthocoronavirinae of the coronavirus family (Nidovirales) and mainly cause respiratory and gastrointestinal infections (Xie and Chen, 2020), such as HCoV-OC43, HCoV-NL63, HCoV-229E, HCoV-HKU1, severe acute respiratory syndrome coronavirus (SARS-CoV), Middle East respiratory syndrome coronavirus (MERS-CoV), and the new 2019 coronavirus (SARS-CoV-2/2019-nCoV) (Figure 1) (Xu et al., 2020). Coronaviruses are spherical or other shaped particles of about 100 nm. The genome length is about 30 kb, which is the largest among the known RNA virus genomes. There are different corona-like spikes on the outer envelope of these viruses, similar in shape to a crown. Therefore, these are known as coronaviruses. SARS-CoV-2 is a β-coronavirus. It is a non-segmented single positive-stranded RNA virus. According to the genome structure, the homology between the SARS-CoV-2 and SARS genome is about 79.5%, which is a precise and rapid gene. Mutation detection can provide an important basis for the screening of antiviral drugs. Structurally, according to genome sequencing, 2019-nCoV is approximately 89% identical to bat SARS-like-CoVZXC21, approximately 82% identical to human SARS-CoV, and approximately 50% identical to MERS-CoV (Dong et al., 2020; Wang et al., 2020c). Coronavirus particles mainly contain four structural proteins, namely the larger spike protein (spike, S), the most abundant viral envelope protein (membrane, M), small envelope protein (envelope, E), and phosphorylated nucleoprotein (nucleocapsid, N), in which the trimeric S glycoprotein mediates the connection between the virus and the host receptor. In addition, the S protein also plays an important role in the host tropism and virulence of the virus, so the S protein becomes the most researched target for related vaccines. As of April 5, 2020, the pandemic has accumulatively caused 1,189,773 confirmed cases and 65,428 deaths globally. The World Health Organization (WHO) is deeply concerned about the unprecedented swift global spread and severity of the epidemic in the world (Xie and Chen, 2020). Hence, WHO declared that COVID-19 can be characterized as a pandemic.

Traditional Chinese medicine (TCM) has been established for thousands of years in China’s medical system (Chen et al., 2020b). It has the characteristics of overall vision, syndrome differentiation, and treatment, and has unique advantages in the prevention, treatment, rehabilitation, and health care of various diseases (Liang et al., 2020). At the same time, TCM plays a vital role in enhancing the function of the main active components and reducing their toxicity through cooperative mechanisms (Hu et al., 2019). In addition, more and more evidence has showed that TCM and its effective components can treat many diseases through a variety of targets (Long et al., 2020), such as pulmonary disease (Zeng et al., 2020), liver diseases (Balkrishna et al., 2020), heart diseases (Chuang et al., 2020), renal diseases (Zhang et al., 2020a), and so on. Meanwhile, the application of TCM in COVID-19 was largely inspired by the treatment of SARS, which was caused by an outbreak of SARS-CoV in Guangdong Province, China. The disease spread rapidly in 2003, and the cumulative number exceeded 8,000 globally (Watkins et al., 1993; Peiris et al., 2004). Of note, TCM also played a significant and credible role in the study of COVID-19 prevention and treatment. Generally speaking, the development of COVID-19 can be divided into three stages: mild, severe/critically severe, and convalescent, forming a complete chain. The team of Xiaolin Tong found that the treatment of COVID-19 with TCM did not aggravate the condition of mild patients, the mortality rate of severe/critically severe patients was reduced by more than 80%, and the rate of symptom improvement in rehabilitation patients was also low (Chen et al., 2020a; Song et al., 2020). Thus, this evidence revealed the safety and effectiveness of TCM in the treatment of COVID-19 patients. In this review, we analyzed data associated with exploring the effectiveness and possible mechanism of action of TCM formulas to prevent and treat COVID-19.

## Overview of Virus-Induced Inflammatory Disease—Focus on COVID-19

In the process of coronavirus infection, inflammatory cytokines have a dual role: on the one hand, they can effectively stimulate the activation of the immune response and enable the body to actively fight foreign viruses; on the other hand, they can mediate and amplify the development of systemic inflammation. It leads to an imbalance of immune function and cytokine storm, but this reaction is not only ineffective to pathogens, but also harmful to the body, and eventually leads to acute respiratory distress syndrome (ARDS), and may lead to the death of patients.

When pattern recognition receptors [PRRs; for example, toll-like receptors (TLRs) and RIG-I-like receptors (RLRs)] are expressed on immune and non-immune cell types resident in tissues, invasive pathogens induce acute inflammation [32156572. PRR connection triggers innate immune response, induces inflammation and antiviral gene expression, limits pathogen growth, activates adaptive immune response, and finally solves infection [26035247. Essentially, the ideal inflammatory response must show a balance, that is, once the threat is controlled, it can be appropriately activated against actual threats and self-limiting behaviors (Chen et al., 2020a). Although it has important significance in maintaining homeostasis in normal tissues and limiting pathogen-related diseases, little is known about the regulatory mechanism of this balance.

**FIGURE 2:**
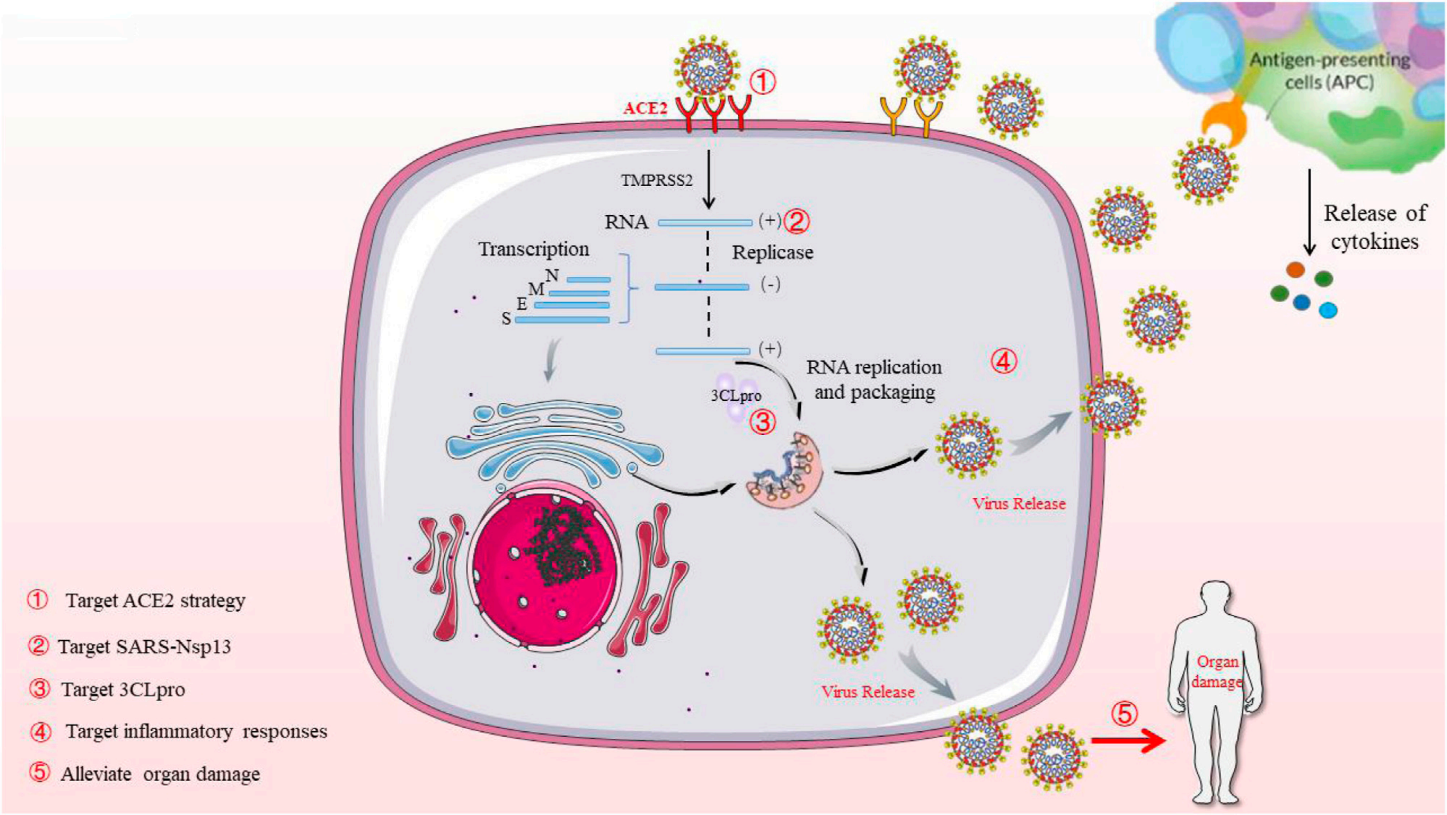
Possible life cycle of SARS-CoV-2 and potential intervention strategy.

The coronavirus family consists of several zoonotic viruses that cause several serious human diseases, including MERS and SARS. At the end of 2019, a novel coronavirus outbreak caused by SARS-CoV-2, resulted in a human disease which was designated by the WHO as COVID-19 (Hanley et al., 2020). Up to now, more than two million people around the world have tested positive for COVID-19, of which more than 200,000 have died of the virus. It is estimated that 15% of patients who were diagnosed with COVID-19 will have serious health complications, while due to the severity of symptoms and high risk of death, about 5–10% of patients need intensive care (3–5%) (Esteves et al., 2020; Gupta et al., 2020). Generally speaking, typical symptoms of COVID-19 include dry cough, dyspnea, fever, fatigue, myalgia, and pneumonia. In most severe cases, complications can occur, such as acute respiratory distress syndrome, which eventually leads to death (Burgueño et al., 2020). SARS-CoV-2 can cause COVID-19 to enter human cells through binding the spike protein and membrane to the aminopeptidase angiotensin-converting enzyme 2 (ACE2) (Kessler and Schunkert, 2020). Meanwhile, more and more evidence shows that inflammatory responses play a vital role in the process of COVID-19. The rapid viral replication and cell destruction of SARS-CoV-2 can recruit monocytes and macrophages, and induce the release of chemokines and cytokines. The SARS-CoV-2 virus and the SARS-CoV-1 coronavirus share many characteristics in common, the latter of which caused a pandemic in 2002–2003 (Sriram and Insel, 2020). Common features include about 80% of the common sequence identity in the virus genome, the infected tissue range, the mortality rate of ARDS, and its cell receptor ACE2 (Zhou et al., 2020a; Zhou et al., 2020b). Meanwhile, this mechanism subsequently leads to a massive influx of neutrophils and monocytes/macrophages, leading to the massive production of pro-inflammatory cytokines, which can destroy lung tissue (i.e., acute respiratory distress syndrome, pneumonia). Specific Th1/Th17 cells may be activated and cause increased inflammation [32417709]. On account of their structural similarity, information associated to SARS-CoV-1 helps to establish hypotheses for the treatment of SARS-CoV-2, which including the re-approval of pharmacology for use in humans. Given the remarkable success of TCM in preventing SARS-CoV-1, TCM may become a key measure to prevent and treat COVID-19 (Figure 2).

## Conventional Treatment of TCM in SARS-CoV Patients

Coronavirus (CoV) belongs to an enveloped positive strand RNA virus in the coronaviridae family (Di Mascio et al., 2020). In the past two decades, CoV has caused two pandemics: SARS infected 8,098 people, with a mortality rate of about 10.5%. MERS infected 2,519 laboratory confirmed cases, with a mortality rate of 34.4% (Gordon et al., 2020). At present, the treatment of SARS and MERS in modern medical clinics includes oxygen inhalation, serotherapy, and antiviral and anti-inflammatory treatment. However, coronavirus-specific antiviral drugs have not yet entered clinical trials, and there are some obvious side effects. At this point, the focus is on compounds with broad antiviral activity and medicine developed for other therapeutic purposes, which also have anti-coronavirus effects.

Related TCM research shows that pulmonary interstitial lesions can be treated according to the principle of removing blood stasis and promoting blood circulation, and can be treated with ligustrazine injection, angelica, and other drugs (Xiao et al., 2003). During the outbreak of SARS in 2003, the State Administration of TCM and the Ministry of Health of the Republic of China announced six prescriptions of TCM to prevent SARS infection. It is worth mentioning that *yiyiren, jiegeng, huangqi, baijiang,* and *gancao* played an important role and acquired prominent effects at that time [16437747. On the other hand, Wen et al. confirmed that six phytoextracts from *ciwujia, longdan, shuyu, juemingzi,* and *sangjisheng* could confer effective anti-SARS-CoV activity by inhibiting SARS-CoV replication. Two compounds from *gouji* (designated as CBE and CBM) also inhibited the 3-Chymotrypsin-like protease (3CLpro) of SARS-CoV (Carroll and Fischer, 1997; Wen et al., 2011).

**FIGURE 3:**
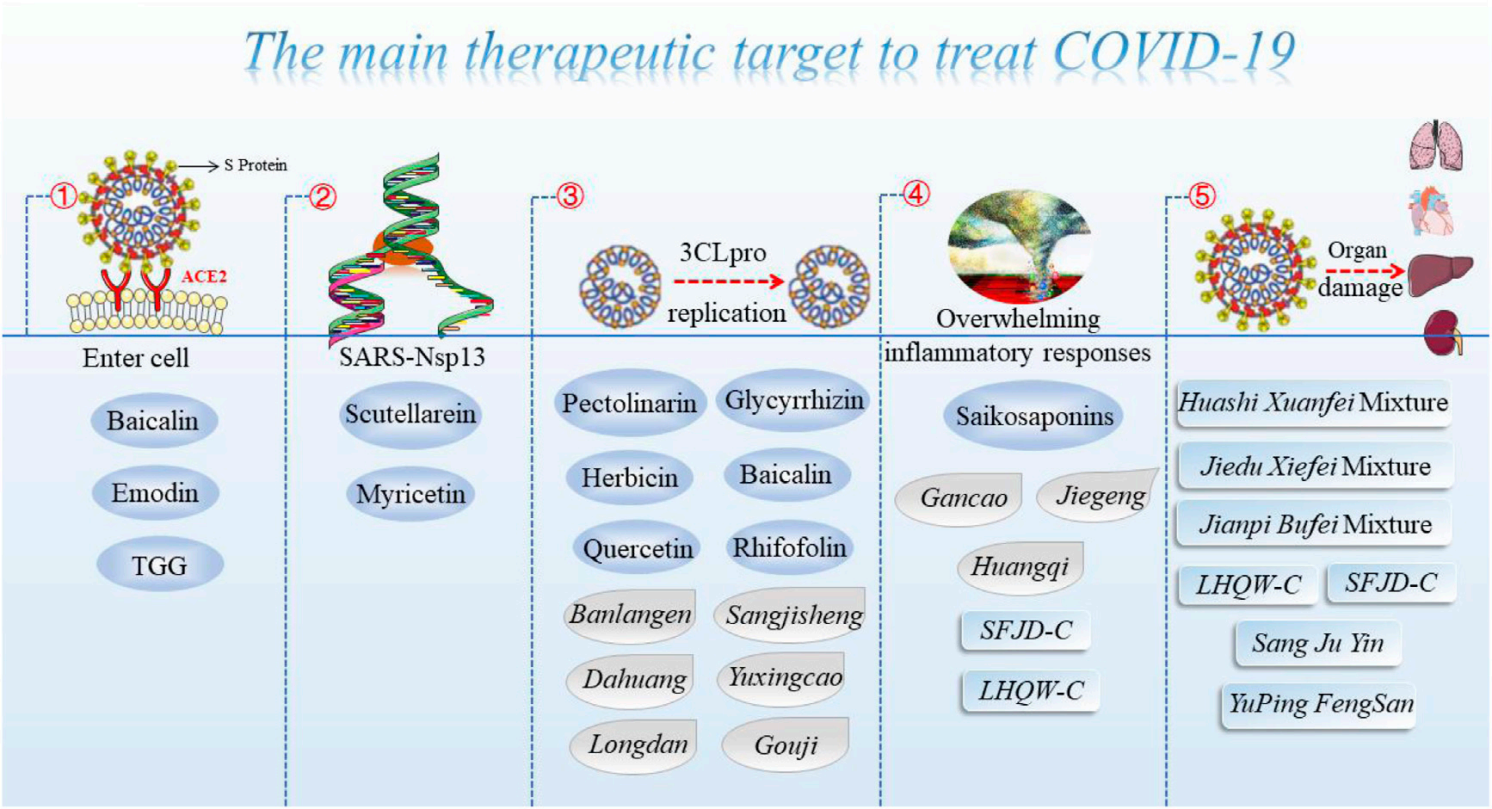
The main therapeutic target to treat COVID-19 (target ACE2 strategy, SARS-Nsp13, 3CLpro, inflammatory responses, and organ damage).

On the other hand, the clinical benefits of TCM seem to be supported by laboratory research to prevent COVID-19. Xi et al. found that in the recovery stage, *banxia,* and *chenpi* were only identified in the list of commonly used herbs, in which patients may have white and thin sputum after recovery. *Banxia* and *chenpi* are the main ingredients of the herbal formula *Erchen* decoction, which is usually used to remove phlegm dampness (Xi and Gong, 2017). Of note, glycyrrhizin, the most effective active component of TCM, effectively inhibits the replication of SARS virus clinical isolates, according to a compelling study published in the Lancet (Cinatl et al., 2003). It serves as one of the antiviral herbal treatments approved by the China Food and Drug Administration (SFDA). Many studies have found that these herbal remedies could inhibit the attachment, entry, and replication of viruses that have previously been used against COVID-19 (Chen et al., 2004; Ang et al., 2020) (Figure 3).

## The Anti-Novel Coronaviral Compound From TCM—Anti-Inflammatory Responses

Natural products derived from TCM are still a rich source of new therapies to treat various diseases. Scientists have made great efforts to identify herbal formulations of various ingredients in TCM with anti-SARS-CoV activity. Simultaneously, although the mechanism of action has not been elucidated, various TCM anti-coronavirus preparations have been identified from TCM. Moreover, Luo et al. studied a total of 26 COVID-19 treatment protocols issued in Chinese provinces and cities and 8 core compatibility and 10 new prescriptions to prevent and treat COVID-19 were identified and proposed. Among them, the most commonly used TCM are *gancao, huangqin, kuxingren, jinyinhua, lianqiao,* and other antipyretic, cough, and phlegm, as well as dehumidification herbs. Most of these TCM have anti-inflammatory, anti-viral, immune regulation, anti-tumor, anti-pyretic and anti-spasmodic, and anti-asthmatic effects (Fan et al., 2020). In the following, we summarized some compounds extracted from TCM that have a certain prevention effect on COVID-19, in order to meet the challenge of conventional treatment of TCM in SARS-CoV patients followed by corresponding potential solutions.

### Single Active Component From TCM–Single Targets Therapy

#### Flavonoids

Baicalin is the biologically active form of *huangqin*, and the major bioactive ingredient of which is flavonoid (Zhao et al., 2020). Su et al. found that the main components of *huangqini* are baicalin and baicalein, and they are novel inhibitors of the COVID-19 3CL protease, which can effectively inhibit the replication of COVID-19 at the cellular level. Moreover, combined with cytotoxicity and viral replication inhibitory activity, baicalein’s cellular antiviral effect of flavonoids is better than most reported compounds. Baicalin tablets have been used as adjuvant treatment for acute, chronic, or persistent hepatitis in China, and baicalein has completed the first phase of clinical trials, and is currently in the second phase of clinical trials against influenza-related symptoms; one decoction was used to treat a new coronavirus infection. The patient’s Chinese medicine or Chinese patent medicine (such as *Qingfei Paidu* decoction and *Shuanghuanglian* oral liquid) also contained *huangqin* (Islam et al., 2020). Of note, ACE2 is the receptor of coronavirus SARS-CoV-2, TCM with the ability of targeting ACE2 is expected to prevent the infection of SARS-CoV-2 [32333601. Deng et al. found that the interaction of the SARS-CoV S protein and ACE2 could be markedly inhibited by baicalin (Deng et al., 2012). But the anti-SARS-CoV activity of these compounds remains to be evaluated.

Quercetin is a plant-derived flavonoid abundant in *gegen*. Related research exhibited that quercetin could inhibit the proliferation and metastasis of some lung diseases (da Silva Araújo et al., 2020; Dong et al., 2020). Epigallocatechin gallate and gallocatechin gallate (GCG), have various biological activities, for instance anti-obesity, anti-bacterial, anti-cancer, anti-oxidative, and cholesterol and triacylglycerol-lowering activity (Wu et al., 2019). Of note, 3-Chymotrypsin-like protease (3CLpro) is of critical importance in virus replication, so it is often a medicine target for the development and treatment of COVID-19 (Zhang et al., 2020b). The results revealed that the purified 3CLpro was used for the inhibition and kinetic analysis of seven kinds of flavonoids, and quercetin displayed a fine inhibitory effect on 3CLpro with an IC50 value of 73 μM (Nguyen et al., 2012). What is more, GCG was the best inhibitor of 3CLpro *in vitro*. The relationship between the structure and inhibitory activity of the seven flavonoids was also studied. GCG revealed many hydrophobic and H-bond interactions of amino acid residues in the active site pocket of 3CLpro (Nguyen et al., 2012).

Herbacetin was extracted from *jinjingcao* (Li et al., 2019). Like other flavonoids, oxalin and its derivatives have nonspecific effects on membrane structure and enzyme activities associated with the occurrence and development of cancer (Péter Zomborszki et al., 2019). Rhoifolin is one of the main flavonoids derived from *chenpi*, which has antioxidant and antihypertensive effects (Liao et al., 2019). Pectolinarin, a major flavonoid, was identified as an active compound in *daji* (Wang et al., 2019). Interestingly, Jo et al. established a flavonoid library and systematically studied the inhibitory compounds of SARS-CoV 3CLpro by the FRET method. Results showed that herbicin, rhifofolin, and pectolinarin were the best choices. The combination of the flavonoids was independently proven by a fluorescence method based on tryptophan. In addition, it is expected that the presence of the additional 8-hydroxyl group of gloxopterin will achieve high binding affinity near the S1 and S2 sites. It is predicted that the carbohydrate groups of pectolinarin and rhoifolin occupy S1 and S2 sites, which is another way that glycosylated flavonoids have a high affinity for SARS-CoV 3CLpro (Jo et al., 2020). This study indicated that the combination of biochemical analysis and docking prediction may help develop better inhibitory flavonoid derivatives from various flavonoid scaffolds.

Myricetin is a natural flavonoid rich in *zhongyao.* It can exert anti-inflammatory, anti-oxidant, and anti-cancer effects (Kang et al., 2020). Scutellarein is a flavone present in *huangqin* which can exert a higher bioavailability compared with scutellarin (Li et al., 2020c). Helicase protein is also served as a potential target for the development of anti-HCoV (human coronavirus) preparations. Helicase SARS-CoV Nsp13 (SARS-Nsp13) plays a critical role in catalyzing the melting of double-stranded oligonucleotides into single-strands in an NTP-dependent manner. Importantly, SARS-Nsp13 has been identified as an ideal target for the development of anti-viral drugs because of its sequence conservation and indispensability in all CoV species (Jia et al., 2019). Yu et al. suggested that myricetin and scutellarein can effectively inhibit the SARS-CoV helicase protein *in vitro* via affecting the ATPase activity instead of relaxing nsP13 activity. Additionally, myricetin and scutellarein show good safety for the lungs because they have no cytotoxic activity against normal breast epithelial MCF10A cells (Yu et al., 2012). Thus, this study demonstrates for the first time that selected naturally occurring flavonoids, including myricetin and scultellarein may be chemical inhibitors of SARS-CoV.

On the other hand, MERS-CoV is a zoonotic virus that spreads between humans and animals. It leads to high mortality from MERS. Jo et al. used the flavonoid library to detect inhibitory compounds against MERS-CoV 3CLpro. As a result, it has been found that isobavachalcone, helichrysetin, herbacetin, and helichrysetin block the enzymatic activity of MERS‐CoV 3CLpro, and these activities have also been independently confirmed by a fluorescence method based on tryptophan. Furthermore, the induced‐fit docking analysis showed that S1 and S2 sites play a role in the interaction with flavonoids. The experimental and computational studies have shown that flavonol and chalcone are ideal scaffolds for binding to the catalytic site of MERS-CoV 3CLpro. We also infer that certain flavonoid derivatives with hydrophobic or carbohydrate moieties possess good inhibitory effects (Jo et al., 2019). Therefore, flavonoids with these properties can be used as templates for the development of effective MERS-CoV 3CLpro inhibitors.

#### Terpenoids

Ginsenoside-Rb1 is the major active ingredient from *renshen*, which has been demonstrated to cause a wide range of biological activities, possibly through anti-stress, anti-oxidative, anti-inflammatory, anti-apoptosis, and anti-depressive effects *in vivo* and *in vitro* (Shi et al., 2020). Wu et al. conducted a large-scale screening of existing drugs, natural products, and synthetic compounds (>10,000 compounds) to identify effective anti-SARS-CoV drugs by a cell-based assay of SARS virus and Vero E6 cells. They revealed that ginsenoside-Rb1 could inhibit SARS-CoV replication at non-toxic concentrations (Wu et al., 2004). Saikosaponins was the major triterpenoid saponins derived from *chaihu* with valuable pharmacological activities. These active components have several immunomodulatory functions, including anti-inflammatory, anti-bacterial, antiviral, and anticancer effects (Sinha et al., 2020). Cheng et al. found that saikosaponins had an effective which can inhibit the viral cellular entry, adsorption, and penetration (Wu et al., 2004).

#### Glycosides

Tetra-O-galloyl-β-D-glucose (TGG) from *wubeizi,* is composed of six galloyl moieties in the glucose core, which strongly inhibits pancreatic lipase activity and adipocyte differentiation in 3T3-L1 cells (Ihn et al., 2019). Yi et al. identified the anti-SARS-CoV activities of TGG, which were confirmed through using a wild-type SARS-CoV infection system. TGG showed significant anti-SARS-CoV activity, the effective concentration was 50%, the concentration was 4.5 μM, and the selective index was 240.0. Additionally, TGG could inhibit the interaction of the SARS-CoV S protein and ACE2 (Yi et al., 2004). Therefore, TGG was potentially useful for the drugs inhibiting SARS.

#### Anthraquinone

Emodin, from the genus *dahuang,* is the main active ingredient of rhubarb. It is an anthraquinone, which displayed various biological effects, such as antibacterial, anti-inflammatory, and anti-viral activities (Li et al., 2020b). Ho et al. found that emodin significantly blocked the interaction between the S protein and ACE2 in a dose-dependent manner. It also inhibited the infectivity of S protein-pseudotyped retrovirus to Vero E6 cells (Ho et al., 2007). In addition, it has previously been shown that the open reading frame 3a of SARS-CoV forms a cation-selective channel, which can be expressed in infected cells and then participated in virus release. It is expected to inhibit viral release to inhibit the ion channels formed by the 3a protein of drugs. Emodin has been shown to inhibit the 3a ion channel of coronavirus HCoV-OC43 and SARS-CoV and the viral release of HCoV-OC43, and its K1/2 value is about 20 μM (Schwarz et al., 2011). These findings indicated that emodin may be considered as a potential main therapeutic agent for SARS (Table 1).

**TABLE 1:** TCM herbal extracts or TCM-derived compounds with anti-HCoV activity.

TCM	Model of action	Ref. (PMID)
Glycyrrhizin	The inhibitory role of virus attachment, entry, and replication	12814717
Baicalin	The inhibitory role of the 3CLpro of SARS-CoV and interaction of SARS-CoV S protein and ACE2	32333601 22136493
Quercetin, GCG, herbicin, rhifofolin, and pectolinarin	The inhibitory role of the 3CLpro of SARS-CoV	31724441 31020684 22350287
Scutellarein	The inhibitory role of the SARS-CoV helicase protein	22578462 31131400
Herbacetin, isobavachalcone, quercetin 3‐β‐d‐glucoside, and helichrysetin	The inhibitory role of the enzymatic activity of MERS‐CoV 3CLpro	31131400
Ginsenoside-Rb1	The inhibitory role of SARS-CoV replication	15226499
Saikosaponins	The inhibitory role of viral cellular entry, adsorption, and penetration	32345124
TGG	The inhibitory role the interaction of SARS-CoV S-protein and ACE2	15452254
Emodin	The inhibitory role of the S protein and ACE2 interaction	21356245
Six phytoextracts from ciwujia, longdan, shuyu, juemingzi, and sangjisheng	The inhibitory role of SARS-CoV replication	9039785
Gancao, huangqin, kuxingren, jinyinhua, and lianqiao	The anti-inflammatory, anti-viral, immune regulation, anti-tumor, anti-pyretic and anti-spasmodic, and anti-asthmatic effects	16437747
Banxia and chenpi	In the recovery stage	32247022
Gouji	The inhibitory role of the 3CLpro of SARS-CoV	9039785
Banlangen	The inhibitory role of the 3CLpro of SARS-CoV	16115693
Dahuang	The inhibitory role of the 3CLpro of SARS-CoV	20103835
Yuxingcao	The inhibitory effects on SARS-CoV 3CLpro and RNA-dependent RNA polymerase (RdRp)	18479853

### Multi-Components From TCM—Multiple Targets Therapy

#### Banlangen

*Banlangen* is a kind of common Chinese medicine to treat colds. It has long been used as a TCM for colds, fever, and influenza, especially for the treatment of SARS and H1N1-influenza (Cao et al., 2019). Previous phytochemical research of *banlangen* have led to the isolation of many natural components, including indirubin, indigo, indican (indoxyl--d-glucoside), -sitosterol, -sitosterol, and sinigrin, etc. (Xi et al., 2020). Lin et al. confirmed that cleavage assays with 3CLpro showed that the IC_50_ values of *banlangen* extract, indigo, sinigrin, aloe emodin, and hesperetin are in the micromolar range. In the cell-based assay, sinigrin (IC_50_: 217 μM) was more effective than indigo (IC_50_: 752 μM) and beta-sitosterol (IC_50_: 1210 μM) in blocking the cleavage processing of 3CLpro. In the cell-based assay, there were only two phenolic compounds, aloe emodin, and hesperetin, that could dose-dependently inhibit the cleavage activity of 3CLpro, in which the IC_50_ was 366 μM for aloe emodin and 8.3 μM for hesperetin (Lin et al., 2005). It suggested that *banlangen* has anti-SARS coronavirus 3C-like protease effects, which might be a potential strategy for treating COVID-19.

#### Dahuang

*Dahuang*, a well-known TCM and food, is usually used to treat various diseases and adverse conditions, such as high fever and abdominal pain [31366111. Its main active components are anthraquinones. A previous study confirmed that *dahuang* extracts possess anti-SARS coronavirus effects (Shi et al., 2020). Luo et al. demonstrated that *Rheum palmatum* L. had a high level of anti-SARS-CoV 3CLpro activity. The IC_50_ was 13.76 ± 0.03 μg/ml and the inhibition rate was up to 96% (Luo et al., 2009). In conclusion, *dahuang* extracts have a high level of inhibitory activity against 3CLpro, indicating that extracts from *dahuang* may represent a potential therapeutic agent for SARS.

#### Yuxingcao

*Yuxingcao* has been used as a TCM to treat a variety of ailments in Asian countries (Liu et al., 2020), and it can effectively treat pneumonia, refractory hemoptysis, infectious disease as well as malignant pleural effusion. Relevant data revealed that *yuxingcao* water extract could obviously and dose-dependently stimulate the proliferation of mouse splenic lymphocytes. In terms of the anti-viral aspect, *yuxingcao* showed vital inhibitory effects on SARS-CoV 3CLpro and RNA-dependent RNA polymerase (RdRp) (Lau et al., 2008).

## TCM Formula in the Treatment of COVID-19 Patients—Multiple Targets Therapy

SARS is an acute respiratory disease that first emerged in 2002. The current SARS-CoV-2 that causes COVID-19 has proved that our preparedness for emerging respiratory viral infections is not enough. TCM exerted a vital role in the treatment of SARS (Lau et al., 2005). According to the quasi-randomized controlled trial involving 654 SARS patients, Liu et al. found that the combination of comprehensive TCM and Western medicines can improve symptoms, quality of life, lung infiltrate and absorption, and reduce corticosteroid dosage (Liu et al., 2006). Therefore, it is possible that TCM could have a potential therapeutic role in COVID-19, which is also a type of SARS. What is more, the guidelines for the diagnosis and treatment of COVID-19 in Hubei province (Sixth Edition) have been developed and released, which also highlights the important role of TCM formulations in the treatment of COVID-19 patients (Table 2) (Yu et al., 2020). Thus, we listed the possible value of the relevant TCM formulations in the prevention of COVID-19.

**TABLE 2:** TCM prescription for treating patients with COVID-19.

TCM prescription	Composition	Model of action
Sang Ju Yin and YuPing FengSan	Sang Ju Yin [made with chrysanthemum, mulberry leaf, and 6 other herbs], Yu Ping Feng San [made with Astragali radix, Astragalus membranaceus, Atractylodes macrocephala, and Saposhnikoviae Radix]	Improved the symptoms and quality of life
Xiang Sha Liu Junzi Tang and Li Zhong pill	Xiang Sha Liu Junzi Tang [made with Citri Reticulatae Pericarpium, Codonopsis Radix, Astragali Radix praeparata cum melle, Poria Sclerotium, Agastachis Herba, Amomi Fructus], Li Zhong pill [made with Pinelliae Rhizoma Praeparatum, Citri Reticulatae Pericarpium, Codonopsis Radix, Astragali Radix praeparata cum melle, Atractylodis Macrocephalae Rhizoma, Poria Sclerotium, Agastachis Herba, Amomi Fructus, Glycyrrhizae Radix, and Rhizoma]	In the recovery stage
Angong Niuhuang pill, Zhi Bao Dan, and Su He Xiang pill	Angong Niuhuang pill [made with Calculus Bovis, Radix Curcumae, rhinoceros horn, Moschus, Rhizoma Coptidis, Radix Scutellariae, Fructus Gardeniae, Cinnabaris, Margarita, Broneolum Syntheticum, and realgar], Zhi Bao Dan [made with Raw black rhinoceros (buffalo horn), raw tortoiseshell, amber, cinnabar, realgar, Niuhuang, borneol, musk, benzoin, gold foil, silver foil], Su He Xiang [made with Styrax, benzoin, borneol, buffalo horn concentrated powder, musk, sandalwood]	In the serious stage
Huashi Xuanfei mixture, Jiedu Xiefei mixture and Jianpi Bufei mixture	Huashi Xuanfei mixture [made with Schizonepeta, peucedana, Platycodon grandiflorum, Stemona, asters, tangerine peel, Houttuynia, mint, poppy shell, liquorice], Jiedu Xiefei mixture [made with notopterygium root, radix scrophularia, Scutellaria, bone skin, mulberry skin, rhubarb, Mangxiao, liquorice], Jianpi Bufei mixture [made with Ginseng, astragalus, beiwuwei, aster, mulberry white skin, prepared rehmannia]	Have significantly improved respiratory symptoms such as fever, cough, fatigue, and digestive tract symptoms
The Lung-toxin Dispelling Formula No. 1	Jingjie, jinyinhua, lianqiao, xuanshen, zaojiaoci, kuxingren, fengfang, gancao, and renshen	Has therapeutic value and no observed side effects in with severe/critical COVID-19 patients
Lianhua Qingwen capsule	Zhimahuang, xingren, lianqiao, bohe, banlangen, yuxingcao, guanzhong, jinyinhua, shigao, dahuang, guanghuoxiang, hongjingtian, gancao	Plays a role in immune regulation, symptom improvement, and anti-inflammatory effects in the treatment of COVID-19
Shufeng Jiedu capsule	Huzhang, lianqiao, banlangen, chaihu, maoweicao, mabiancao, lugen, and gancao	The therapeutic mechanisms involve a variety of biological processes, such as viral interactions

Importantly, severe inflammatory responses can be attributed to the deaths of SARS-CoV, MERS-CoV, or COVID-19 patients. As a result, anti-inflammatory drugs may reduce the rates of severity and mortality (Lu, 2020). Interestingly, it was discovered that *shuanghuanglian* effectively inhibited the stimulation of cytokines (IL-1β, IL-6, TNF-α, IFN-γ) and chemokines (MIP-1α, MIP-1β, and MCP-1) in peripheral blood mononuclear cells (PBMCs) induced by staphylococcal toxic shock syndrome toxin 1 (TSST-1) (Chen et al., 2002; Science CAo, 2020). Meanwhile, Poon et al. found that *Sang Ju Yin* and *Yu Ping Feng San* may have beneficial immunomodulatory effects on preventing viral infections, including SARS-CoV. “Clinical study on the treatment of COVID-19 with Chinese medicine” has made progress recently. Three new anti-coronavirus pneumonia Chinese medicine preparations have been approved by the Zhejiang Provincial Drug Administration as hospital preparations, laying a better foundation for further prevention and treatment of novel coronavirus pneumonia. Three kinds of TCM preparations against COVID-19 are jointly used by famous doctors of national medicine in the hospital, and national and provincial Chinese medicine. The three kinds of TCM formula that have been developed are: New Crown Pneumonia No. 1 prescription-*Huashi Xuanfei* Mixture (treatment of common type) New Coronary Pneumonia), New Coronary Pneumonia No. 2-*Jiedu Xiefei* Mixture (Treatment of Severe New Coronary Pneumonia), New Coronary Pneumonia No. 3-*Jianpi Bufei* Mixture (Treatment of New Coronary Pneumonia). Of note, these three prescriptions are being used in nine new coronavirus pneumonia designated hospitals in the province. The results show that: after using TCM, patients have significantly improved respiratory symptoms such as fever, cough, fatigue, and digestive tract symptoms caused by antiviral drugs. Some severely ill patients become mild after receiving treatment.

The Lung-toxin Dispelling Formula No. 1 (祛肺毒一号方), known as Respiratory Detox Shot (RDS), which is based on the medicinal value of the TCM theory, classical TCM prescription, and clinical practice. There are nine TCM ingredients in RDS: *jingjie*, *jinyinhua*, *lianqiao*, *xuanshen*, *zaojiaoci*, *kuxingren*, *fengfang*, *gancao,* and *renshen*. The nine TCMs have been proven to be effective in preventing and treating acute respiratory infection, common cold, and influenza in clinical practice. Lately, a clinical trial evaluating the efficacy of RDS in severe/critical COVID-19 patients found that RDS had therapeutic value and no observed side effects (Li et al., 2020a)**.** Zhang et al. confirmed that RDS mainly acted on the kidney-urinary bladder, lung-large intestine, and stomach-spleen meridians, while other zang-fu viscera were strategically covered by all nine components. Under the background of the meridian theory of TCM, multiple components and objectives of RDS contribute to the health and elimination of pathogens. (Zhang et al., 2020b). Thus, this has a universal effect on the early control and prevention of COVID-19.

The *Lianhua Qingwen* capsule (LHQW-C) is a TCM prescription composed of *zhimahuang*, *xingren*, *lianqiao*, *bohe*, *banlangen*, *yuxingcao*, *guanzhong*, *jinyinhua*, *shigao*, *dahuang*, *guanghuoxiang*, *hongjingtian*, and *gancao* (Zhao et al., 2014). LHQW-C has been used to treat conventional seasonal influenza for decades. LHQW-C is found to affect or inhibit all parts of human influenza A virus in patients infected with human influenza A virus in different periods (Ding et al., 2017). Furthermore, Li et al. found that LHQW-C significantly inhibited SARS-CoV-2 replication in Vero E6 cells and significantly reduced pro-inflammatory cytokine (TNF-α, IL-6, CCL-2/MCP-1, and CXCL-10/IP-10) production at the mRNA levels. Furthermore, LHQW-C caused abnormal particle morphology of virus particles in cells (Runfeng et al., 2020). One eligible study focused on the anti-viral and anti-inflammatory activities of LHQW-C for treating COVID-19 or the suspected cases under medical observation, which published by the team of the outstanding academician Nanshan Zhong. Of note, for suspicious or mild cases, early treatment was essential. Relevant data showed that LHQW-C could be used as a preventive measure for the patients in the medical observation period (Runfeng et al., 2020). In addition, the clinical evidence confirmed that LHQW-C has quite the curative effect when combined with a Western medicine scheme, and no obvious adverse reaction. It can not only improve the clinical symptoms including cough, fatigue, fever, sputum, shortness of breath, chest tightness, poor appetite, but it also can control the progress of the upper respiratory tract infection and reduce the transition from light to severe, and shorten hospitalization time, which is suitable for ordinary type and suspected COVID-19 patients (Wang et al., 2020b). Therefore, accumulating evidence from bioinformatics, pharmacodynamics, and clinical findings suggests that LHQW-C plays a role in immune regulation, symptom improvement, and anti-inflammatory effects in treating of COVID-19.

The *Shufeng Jiedu capsule* (SFJD-C) is composed of *huzhang, lianqiao, banlangen, chaihu, maoweicao, mabiancao, lugen,* and *gancao.* It was mainly used to treat acute upper respiratory tract infections previously (Ying et al., 2017). Xiao et al. found that SFJD-C combined with arbidol had good efficacy in the treatment of COVID-19 and no drug-related adverse events were reported. What is more, there were critical improvements in CD4^+^ and CD8^+^ T cell subpopulations, white blood cell and CT imaging, antipyretic time, the disappearance time of dry cough, nasal congestion, fatigue, pharyngeal pain, and diarrhea for 2019-nCoV (Luo et al., 2009; Fung et al., 2011). At the same time, the study about a drug-component disease-target network analyzed the potential targets and mechanisms of SFJD-C for treating COVID-19. The results indicated that the therapeutic mechanisms involved various biological processes, such as viral interactions. And there were various cytokines, such as IL-6, ALB, and MAPK3 (Ying et al., 2017). In patients with COVID-19 infection, a huge number of cytokines may be related to the rapid onset of acute respiratory distress syndrome (ARDS), single or multiple organ failure, and even eventual death.

## Prospect of Application of TCM in COVID-19

TCM has obvious advantages in preventing and treating COVID-19. A variety of TCM have antiviral, anti-inflammatory, immune regulation effects, and low toxicity and side effects. In particular, some TCM have a two-way regulatory effect on the immune system. They enhance the body’s immunity through improving immunity and protecting all organs. It can also reduce an excessive immune response and alleviate the damage caused by inflammatory storm. However, TCMs fail to occupy an important position in the international market, because of the lack of reliable quality standards of TCM, and the fact that its specific mechanism is not clear. Based on this, it is urgent for us to find out the effective components in TCM, so that the specific mechanism of its effect can be explored. Therefore, we may be able to screen some effective components of TCM and study their mechanism of action. Thus, we could develop some drugs that can treat COVID-19. On the other hand, for TCM prescriptions, we can determine the quality standard and the proportion of each TCM to better treat COVID-19. In particular, a TCM formula could treat COVID-19 with multiple targets at the same time. Luo et al. found that *gancao, huangqin, kuxingren, jinyinhua, lianqiao,* and other herbs are the most frequently used in TCM, and these showed anti-inflammatory, anti-viral, immune regulation, anti-tumor, anti-pyretic and anti-spasmodic, and anti-asthmatic effects (Fan et al., 2020). Clinical studies have shown that LHQW-C significantly improved fever, cough, fatigue, and shortness of breath in suspected and confirmed COVID-19 cases, and reduced the proportion of suspected cases turning to severe disease. What is more, the over-expression of inflammatory cytokines (IL-6, TNF-a, MCP-1, and so on) caused by SARS-CoV-2 was significantly inhibited by LHQW-C, which exerted a vital role in immune regulation, symptom improvement, and anti-inflammatory effects in treating COVID-19. It also offers a reference and a potential pathway for the development of modern medicine, especially for the development of modern compound preparation to treat COVID-19.

In the last few decades, genome editing technology has been largely used in biomedical research. At the same time, continuous development has improved the efficiency of gene editing, reduced the impact of deviation from the target, and simplified the operating procedures in the organism (Memi et al., 2018). CRISPR/Cas13d is the RNA-directed RNA targeting CRISPR system. Tuan et al. suggest using the system to “chew” the RNA genome of SARS-CoV-2 specifically to limit its ability to regenerate. Cas13d protein and guided RNAs (gRNAs) containing specific complementary spacer sequences with viral RNA genomes were selected to cut the RNA genome of SARS-CoV-2. Moreover, in order to destroy the function of the virus, the researchers specially used the gRNAs to target both the ORF1ab and S genes of SARS-CoV-2. These functional proteins play a vital role in the infection and replication of SARS-CoV-2. If they interfere with the function of one or more of the proteins individually or at the same time and inhibit their activity, they can block the process of SARS-CoV-2 infection or self-replication in the host cell, so as to play a therapeutic role (Nguyen et al., 2020). Therefore, we can reduce the toxicity of natural products by subtracting toxic genes through gene editing. Hence, the natural productions would only protect COVID-19 patients.

**FIGURE 4:**
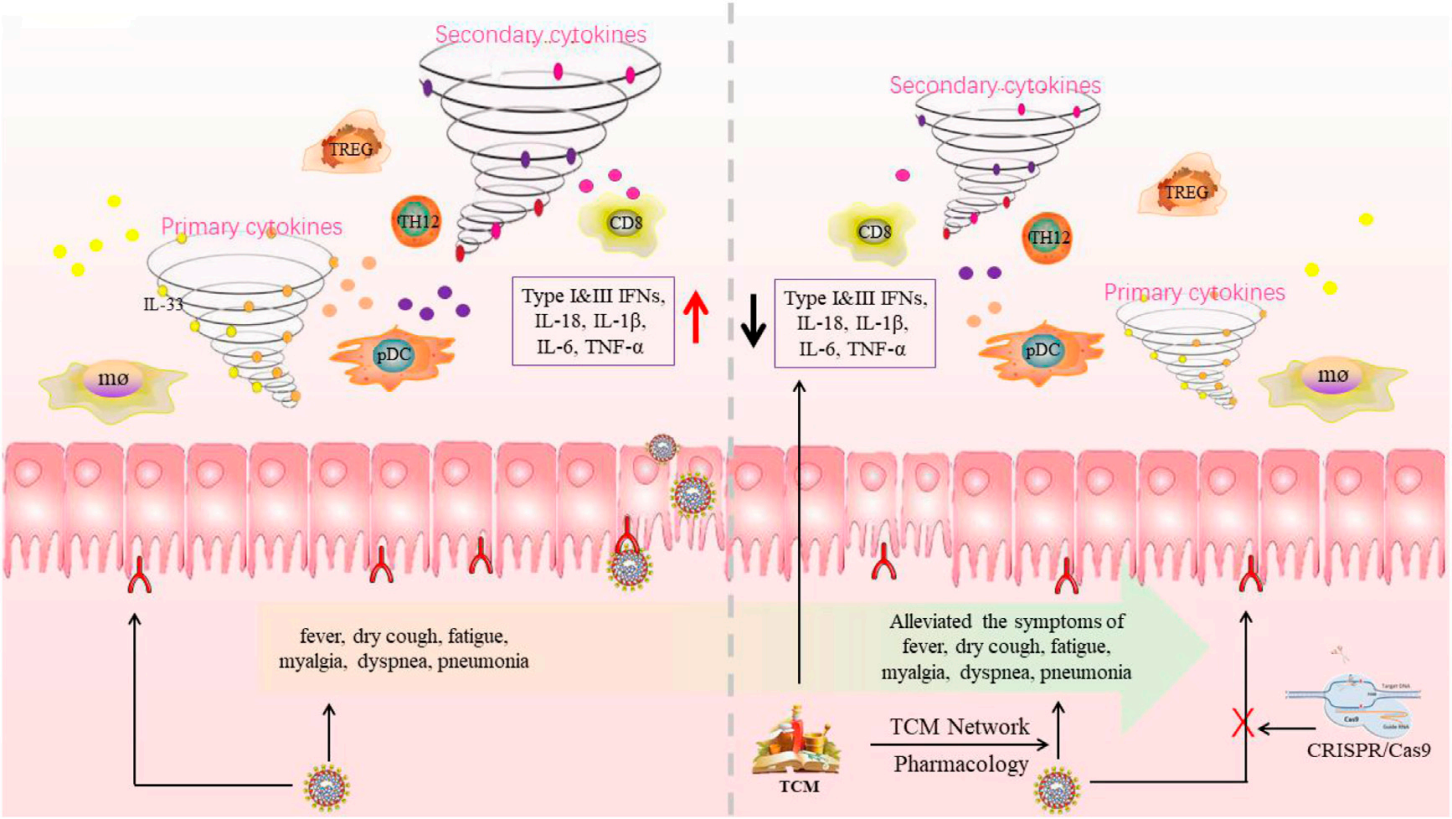
The future expectation of therapeutic methods in the treatment of COVID-19 patients. The researchers will specially use the gRNAs targeting both ORF1ab and S genes of SARS-CoV-2 in order to destroy the function of the virus and the toxicity of natural products by subtracting toxic genes through gene editing. Meanwhile, we can use the TCMSP database to predict and screen the effective active ingredients of related TCM and sort out the ingredients of each prescription respectively by using the network pharmacology method to treat COVID-19.

On the other hand, to further explore the mechanism of TCM treatment of COVID-19. We used the TCMSP database to predict and screen the effective active ingredients of related TCM and sort out the ingredients of each prescription respectively by using the network pharmacology method. We integrated the targets of the common ingredients of the four formulations predicted on the Swiss Target Prediction platform and the targets related to COVID-19 obtained in the GeneCards database into a Venn diagram to screen the common targets. Next, we used the string platform to analyze the protein-protein interaction (PPI) between the common target and ACE2. Then, we analyzed the KEGG pathway and GO enrichment by using the DAVID platform, and studied the interaction between the target protein and HIV protein. On the basis of the above research, the results were visualized using the OmicShare platform and Cytoscape software (Zu et al., 2020) (Figure 4)**.**

## Conclusion

In the search for novel antiviral agents, it is not a new idea to reuse old drugs or relocate experimental drugs, and it was proposed that they should be used to treat other virus infections, such as Ebola epidemic (Sweiti et al., 2017). Meanwhile, it is also recommended to repurpose folk medicines (Habtemariam and Lentini, 2015), while emphasizing cardiovascular medicines (Patanè, 2014). Diseases caused by virus have been a big problem for scientists. For thousands of years, until today, no effective and special drugs have been found in the medical field. However, so far, there are no specific antiviral drugs or vaccines that have been proven to be effective against virus-induced inflammatory diseases, such as COVID-19. Therefore, the focus is on preventive measures and symptomatic treatment. Of note, because COVID-19 can cause systemic organ damage, multi-target therapy may be required besides anti-inflammatory and antiviral drugs during treatment. TCM can protect COVID-19 patients from tissue damage. This protection can be at least partially attributed to the anti-inflammatory, antioxidant, and anti-apoptotic effects of the TCM under investigation. In this context, TCM are being explored to provide patients with supportive, preventive, and rehabilitative care, owing to the characteristics of the multi-target therapy of TCM. Although there is no direct evidence, some uncontrolled studies on TCM show that they may possess a direct efficacy on the virus (Weeks, 2020). This also provides a reference and idea for the development of modern drugs, especially the development of modern compound preparations. In this review, it can also provide drug candidates for medicine research and development of COVID-19 prevention and treatment.

## Abbreviations

ACE2, Angiotensin-converting enzyme 2; ARDS, Acute respiratory distress syndrome; 3CLpro, 3-chymotrypsin-like protease; COVID-19, Coronavirus disease 2019; CoV, Coronavirus; gRNAs, Guided RNAs; IL-1β, Interleukin-1 β; IL-18, Interleukin-18; LHQW-C, lianhua-qingwen capsules; MERS-cov, middle east respiratory syndrome coronavirus; MOF, Multiple organ failure; PBMC, Peripheral blood mononuclear cell; PPI, Protein-protein interaction; RDS, respiratory detox shot; rdrp, RNA-Dependent RNA polymerase; SARS, Severe acute respiratory syndrome; SARS-cov, Severe acute respiratory syndrome coronavirus; SARS-cov-2/2019-ncov, Severe acute respiratory syndrome coronavirus-2/2019 new coronavirus; SFJD-C, shufeng jiedu capsules; TGG, tetra-O-Galloyl-β-D-glucose; TSST-1, Toxic shock syndrome toxin 1.
